# Diagnostic Accuracy of p16^INK4a^/Ki-67 Dual Immunostaining for Detection of High-Grade Cervical Intraepithelial Neoplasia in Women Involved in Cervical Cancer Screening in Georgia

**DOI:** 10.1155/2023/7988323

**Published:** 2023-06-05

**Authors:** Sopio Kakaliashvili-Dzagnidze, Omar Khardzeishvili, Sergo Tabagari

**Affiliations:** ^1^Pathology and Pharmacology Research Department, Medical school, Georgian American University, Tbilisi 0160, Georgia; ^2^David Tvildiani Medical University, Tbilisi 0159, Georgia; ^3^Department of Anatomic Pathology, Faculty of Medicine, Tbilisi State Medical University, Tbilisi 0186, Georgia

## Abstract

**Background:**

Despite the widespread introduction of primary and secondary preventative measures, death rates for cervical cancer are still significantly high among females, especially in developing countries. Pap cytology and human papillomavirus-based screening often lead to unnecessary additional testing. The aim of this study is to analyze diagnostic accuracy of p16^INK4a^/Ki-67 dual immunostaining (DS) in cervical smear for identifying high-grade cervical intraepithelial neoplasia (CIN2+).

**Materials and Methods:**

We studied the diagnostic performance of p16^INK4a^/Ki-67 DS in cervical smear of those women, who enrolled in cervical cancer screening due to abnormal previous screening results and compared it with Pap test results in identifying CIN2+. The reference standard was histopathology results. p16^INK4a^/Ki-67 DS and Pap test results for 162 women and histopathology results for 29 women were available, respectively.

**Results:**

In our study, sensitivity, specificity, positive predictive value, and negative predictive value of p16^INK4a^/Ki-67 DS, irrespective of the morphology of stained cells to detect CIN2+ were 100%, 89%, 85%, and 100% (*p* < 0.01), respectively. The diagnostic accuracy of p16^INK4a^/Ki-67 DS is superior to that of existing cervical screening tests in the detection of CIN2+.

**Conclusion:**

The findings of cervical cancer screening based on Pap cytology highlight the importance of assessing the cost-effectiveness of integrating p16^INK4a^/Ki-67 biomarkers in cervical cancer cytology. Furthermore, these findings emphasize the need to enhance support for preventive programs for cervical cancer in Georgia.

## 1. Introduction

The burden of cancer-related mortality and morbidity can be reduced by implementing efficient tools for the early detection of precancerous lesions. Cervical cancer is the fourth most common cancer among women globally, with an estimated 604,000 new cases and 342,000 death in 2020, among them 90% of the new cases and death occurred in low- and middle-income countries [1]. The existence of precancerous lesions for invasive cervical cancer has been recognized for over a century [2]. Almost all carcinomas of the uterine cervix are derived from precancerous lesions or cervical intraepithelial neoplasia (CIN) [3, 4], but a minority of women with CIN develop cervical cancer [5]. Adoption of Pap test-based screening and The Bethesda System (TBS) categorisation improved cervical cancer burden over decades, but according to studies, Pap test has low sensitivity to detect high-grade cervical intraepithelial lesion [7]. For over 20 years, it has been evident that high-risk human papillomavirus (HR-HPV) causes almost all squamous cell carcinomas of the cervix as well as the vast majority of adenocarcinomas of the cervix [8]. Even though the prevalence of HPV infection is high, only a small number of infected individuals develop cancer [9], since the infection is mostly transient [10].

American Cancer Society and European International Agency for Research on Cancer approved three primary screening approaches for women between 21 and 65 years old: Pap test, HR-HPV DNA test, and the co-testing (Pap test plus HPV test) [11, 12]. Although of population-based Pap test and HPV test implemented in most developing countries, still cervical carcinoma is one of the common cancer of females throughout the world [13] and leading causes of death in many developing countries [1].

According to many studies existent cytological and HPV screening recognize mostly transient cervical lesions, investigation, and treatment of which do not benefit the patient [14], rather not necessary invasive diagnostics and excisional treatments may increase the risk of anxiety and stress in young women, the premature rupture of membranes and preterm delivery [15–17]. Furthermore, the longevity of reproductive years and repeated recruitment of females with abnormal cytologic results, back into the screening program, may affect logistics and financial resources, especially in low-income countries.

Pap test-based cervical cancer screening has been implemented in Georgia since 2008 and the program become national wide since 2011. Screening has been opportunistic for years and since 2022 it has become population-based. The coverage of the national cervical cancer screening program is 23%. HPV vaccination was included in the national vaccination program in 2019. Final-dose vaccine coverage in girls is 22% [13]. There are still few peer-reviewed publications on screening outcomes in Georgia [25].

TBS classification categories precursors of cervical cancer based on Pap test results from negative for intraepithelial lesion or malignancy (NILM) to precancerous lesions of low-grade squamous intraepithelial lesion (LSIL) to high-grade SIL (HSIL) and ultimately invasive squamous cell carcinoma [6]. However, depending on the qualitative and quantitative limitations of the cytology specimen, some equivocal morphological features suggestive of squamous cell abnormality fall under the equivocal category: “atypical squamous cells” (ASCs), which are subdivided into two categories: “atypical squamous cells of undetermined significance” (ASC-US) or “atypical squamous cells, HSIL cannot be excluded” (ASC-H), based on the suspected underlying lesion LSIL or HSIL, respectively [26]. Nuclear atypia and perinuclear cytoskeletal abnormalities are the most specific features of SIL. These simple but important morphological clues of SIL may be difficult to be interpreted due to the subtle overlap of morphology with other non-neoplastic squamous changes. Those changes may be associated with protective and reactive responses to inflammation, hormonal alterations, and colonizing or infectious organisms. Due to the wide spectrum of reactive cytomorphologic changes, criteria are not well-defined and may lack reproducibility [27, 28].

According to the College of American Pathologists and the American Society for Colposcopy and Cervical Pathology sponsored the Lower Anogenital Squamous Terminology (LAST) project recommendations, SIL terminology can be used for histopathology of HPV-associated noninvasive cervical squamous lesions [29]. LSIL corresponds to CIN1, and HSIL corresponds to CIN2+ (CIN2 and CIN3). Meanwhile, for intervention, it needs to differentiate HSIL into CIN2 or CIN3, as management is different among pregnant/non-pregnant women.

There is no corresponding histology terminology for the cytologic ASC category in the existing classification. Histologic classification and terms of cervical precancerous lesions—dysplasia and CIN still used in clinical practice for the management of patients. Inconsistency in cytology versus histology classification, and the use of different classifications cause confusion among specialists and patients.

Pap test has qualitative and quantitative limitations [26]. Due to the wide spectrum of reactive cytomorphologic changes, the criteria of cytology-based Pap test results are not well-defined and may lack reproducibility among pathologists [27, 28, 31]. The reporting of specific non-neoplastic findings is optional and is at the discretion of the laboratories. Cervical precancerous lesion is not a reportable disease, and there is no existing reference laboratory for Pap test results in Georgia to trigger an additional review of cytology results.

Given that, cytology-based screening is subjective, and HPV-based testing only detects infection rather than presence of disease, the introduction of an integrated cytology marker concept can be promising step forward improving cervical cancer screening strategy. Recent studies on various biomarkers have emphasized their significant role in diagnosing precancerous lesions and enabling personalized treatment. Several biomarkers, including p16^INK4a^ and Ki-67 cellular proteins have been extensively studied by various scientists and have shown promising results.

Petry et al. first proposed the concept of p16^INK4a^/Ki-67 dual staining cytology and its role in cervical cancer screening [18]. p16^INK4a^ is a cellular protein that mediates cell-cycle arrest [19]. Normally, transcription of cellular protein p16^INK4a^ is repressed by pRb, the regulator protein of the cell cycle, in tumor cells lacking the pRb function. The p16^INK4a^ transcription is activated and is overexpressed due to the removal of the pRb repression [20]. HR-HPV induced viral oncoprotein E7 disrupts pRb/E2F interaction in the infected epithelial cells, releases active E2F, and induces pRb degradation through a proteasome-dependent mechanism [21]. Inactivation of the pRb induces p16^INK4a^ overexpression, which is an indicator of HR-HPV induced transformation of epithelial cells [22].

The Ki-67 is a proliferation-associated nuclear protein, only detected in dividing cells (G1-, S-, G2-, and M-phase) and not in quiescent cells (G0 phase) makes it an excellent marker for determining the so-called growth fraction of a given cell population [23]. Ki-67 cellular protein has been widely used in the auxiliary diagnosis of cervical precancerous lesions and cancer [30]. Simultaneous detection of cell cycle inhibitory protein p16^INK4a^ and the cell cycle progression marker Ki-67 in the same cell allows for the unequivocal identification of truly HPV-induced oncogenic transformation of the cells [24]. Many published papers show different accuracy of simultaneous expression of dual p16^INK4a^/Ki-67 biomarkers in cervical epithelial cells for detection of cervical precancerous lesions. There are only a few existing scientific papers on cervical cancer screening results from Georgia. The study aimed to evaluate the diagnostic accuracy of p16^INK4a^/Ki-67 DS in cervical cytology to identify high-grade CIN (CIN2+) in women participating in opportunistic cervical cancer screening in Georgia, compare it with Pap test results and determine the role of integration of biomarkers in cytology-based screening.

## 2. Materials and Methods

We performed an analysis of p16^INK4a^/Ki-67 DS and pathomorphological results of cervical material obtained from women participating in an opportunistic Cervical Cancer Screening Program in Georgia, from March 2011 to December 2013. These women were enrolled in the screening program either based on the recommendation of gynecologists or their own decision, following abnormal primary Pap test results. Women for repeated testing applied to nine medical centers, licensed for providing gynecological services in Tbilisi, Georgia: MediClubGeorgia, Medical Center Iunona, Venus Georgia, Medicare Georgia, Caraps Medline, In Vitro, Interclinic, Gudushauri Clinic, and Chachava Clinic. Pap test and p16^INK4a^/Ki-67 DS were offered to the women by recommendation of the gynecologists. Cervical smears of 162 women were stained and analysed for p16^INK4a^/Ki-67 dual immunocytochemistry. Materials of paraffin block for two out of all women were stained by p16^INK4a^ immunohistochemistry (p16 IHC). p16^INK4a^/Ki-67 DS and p16 IHC were conducted in three pathologic laboratories: MediClubGeorgia, Pathology and Anatomical Scientific-Practical Center of Adult and Child Pathology, and Cytogen that had contracts with corresponding clinics with gynecologic departments. Biomarker expression was conducted by a trained and certified specialist, and slides were assessed by three independent pathologists. Pap test results of all women, as well as histopathologic results of biopsy materials of 29 out of all women were available from corresponding clinics. The number of biopsies, as well as IHC was determined upon recommendation of the gynecologists, taking into account the screening algorithm and also the patient's compliance with the doctor's recommendations. We compared p16^INK4a^/Ki-67 DS and Pap cytology results of cervical smears taken at the same time from the cervix of women. Hematoxylin and eosin (H&E) stained histopathology results were used as the reference standard. IHC staining was used in equivocal H&E histopathology results.

Follow-up cytology screening results were obtained from MediClubGeorgia.

Cases were excluded from the study where the following: (1) slides did not meet the minimum squamous cellularity criteria as specified in the Bethesda 2001 Cervical Cytology Classification system for reporting cervical cytology, (2) absence of Pap test results, (3) pregnancy, (4) ongoing chemotherapy, and (5) radical hysterectomy. In our study, p16^INK4a^/Ki-67 dual immunocytochemical examination of cervical smear was performed in parallel with a Pap test.

### 2.1. Collection of Materials

Staining on biomarker expression was performed by a certified specialist, who conducted training on immunocytochemistry diagnostics, in MTM Laboratories, Germany, Heidelberg, 2010. All gynecologists were informed about timing (proliferative phase) and technical procedures regarding taking samples from cervix for immunostaining. The collection of cytologic smears was conducted based on the protocol of the manufacturer for immunostaining. For collection of cervical cytology material, we used a vaginal speculum and a wedge-shaped, broom-like cervical device. Cytology material was collected by rotating the cervical device in the cervix in a clockwise direction 360°, five times. Collected materials were smeared on glass slides. Cell material was collected for immunostaining and Pap staining. Cytologic specimens for immunostaining were fixed with cytological spray fixation reagent containing polyethylene glycol (Merkofix) immediately after sample collection and dried for 20 minutes, after that, the material was transported by special containers to pathology laboratories. Pap testing and H&E histopathologic diagnostics were conducted in contracted laboratories of corresponding medical facilities. Immunohistostaining was conducted on the remaining material of the paraffin block.

### 2.2. Slide Preparation and Immunostaining

Immunostaining of cervical cytology material was performed by using the CINtec PLUS Kit in the pathology laboratory, according to the instructions of the manufacturer (REF 9531, MTM Laboratories). In brief, procedures performed in the following steps: (1) reagent preparation and equilibration at 20–25°C; (2) specimen rehydration; (3) epitopal retrieval; (4) staining; (5) counterstaining; and (6) two-step mounting. For detection of antigens, a primary monoclonal mouse antibody clone E6H4TM directed to human p16^INK4a^ protein and a primary monoclonal rabbit antibody clone 274-11 AC3 directed against human Ki-67 protein were used. Antigen retrieval was performed for 10 minutes a 95°C–99°C in a water bath. Staining was conducted by using Shandon Coverplate™ System (REF 2010-953-009EU). After blocking endogenous peroxidase activity, the slides were incubated for 30 minutes with primary antibody p16^INK4a^/Ki-67 solutions. Visualisation Reagent horseradish peroxidase (HRP) (polymer reagent conjugated with HRP and affinity-purified goat anti-mouse Fab antibody fragments) was applied for 15 minutes. Visualisation Reagent alkaline phosphatase (AP) (polymer reagent conjugated with AP and affinity-purified goat anti-rabbit Fab antibody fragments) was applied for 15 minutes. 3,3′-Diaminobenzidine (DAB) Substrate Chromogen Working Solution (prepared based on instruction just before staining) was applied for 10 minutes and Red Fast Substrate Chromogen Working Solution (prepared based on instruction just before staining) was applied for 15 minutes. Chromogenic visualisation slides were removed from Shandon Coverplate™ gently, and counterstaining by use of alcohol-free hematoxylin was performed. The mounting procedure was conducted in two steps: first, liquid based mounting, with incubation overnight at ambient temperature. Second, after complete drying, slides were incubated in xylene for 1–20 minutes, and a xylene-based mounting medium was used for coverslips the slides. For quality control, there were used positive and negative controls, for which Pap-stained slides with cervical cancer and normal cytology were used with de-staining and re-staining according to the manufacturer's protocol.

Immunostaining of cervical biopsy specimens for the p16^INK4a^ biomarker was performed using the CINtec Histology Kit (REF 9511, MTM Laboratories) according to the instructions of the manufacturer. In brief, antigen retrieval was performed for 10 minutes at 95–99°C in a water bath. After blocking endogenous peroxidase activity, the slides were incubated for 30 minutes with the p16^INK4a^ antibody (clone E6H4) or with the Negative Reagent Control (isotype control antibody), both included in the CINtec Histology Kit. Secondary antibody reagent (polymer-based goat-antimouse antibody fragment conjugated with HRP) was applied for 30 minutes. After the chromogenic visualisation step using the 3,3′-DAB chromogen, slides were counterstained with hematoxylin and coverslipped. For each staining run, a positive control slide containing tissue sections from a cervical biopsy with known positive immunoreactivity for p16^INK4a^ was used to validate the staining procedure.

### 2.3. Interpretation of Results

**Figure 1:**
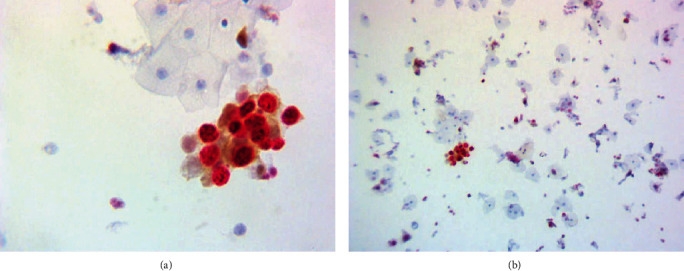
Positive p16^INK4a^/Ki-67 DS cells. (a) Magnification ×400. (b) Magnification ×100.

**Figure 2:**
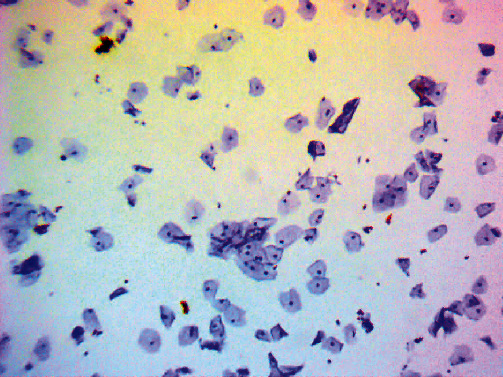
Negative p16^INK4a^/Ki-67 DS cells, magnification ×100.

For the interpretation of p16^INK4a^/Ki-67 DS of cytology slides, a trained cytotechnologist reviewed all cases for the presence of dual-immunoreactive cells. Under light microscopic examination, the presence of more than one cervical epithelial cell in a cluster, irrespective of cell morphology, with a brown cytoplasmic and red nuclear staining were categorised as a positive result of p16^INK4a^/Ki-67 DS (Figure 1). Cases without p16^INK4a^/Ki-67 DS were categorised as negative (Figure 2). After reviewing by a cytotechnologist, all immunostained slides were evaluated by three independent pathologists. Pap tests and histopathology results were collected from the patient's medical records.

**Figure 3:**
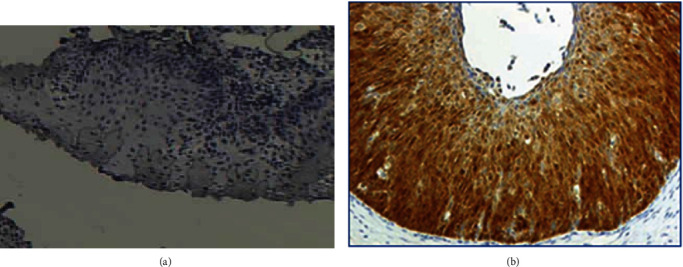
p16 IHC staining. (a) Negative p16 IHC, magnification ×100. (b) Positive p16 IHC, magnification ×100.

For the interpretation of p16 IHC staining, the classification was conducted according to the following parameters: (1) intensity: strong (dark brown color similar to the positive control) versus weak (yellow color significantly lighter than the positive control); (2) extent: diffuse (signal involves >50% of the epithelium) versus focal (<50% of the epithelium); (3) continuity: continuous (staining extends laterally over a significant distance) versus discontinuous (alternating clusters of either positively or negatively stained cells); and (4) location: positive cells reside in the lower third, two thirds, or full thickness of the epithelium. Based on these four parameters, lesions were categorised as a positive and negative pattern (Figure 3). IHC categorisation patterns fulfilled all requirements described in LAST: positive results correspond to strong, diffuse, and continuous immunoreactivity extending from the basal layers upward to more than one-third of the epithelium. Negative results were defined as the total absence of staining or else weak, focal, and discontinuous staining. Based on literature data p16 positive expression corresponds to HSIL, and negative p16 corresponds to <CIN1 [29].

### 2.4. Statistical Analysis

All collected data were entered into the database and underwent statistical analysis. The data were analysed with the program SPSS (IBM Corp. Released 2011. IBM SPSS Statistics for Windows, version 20.0, IBM Corp., Armonk, NY, USA). *χ*^2^ test or Fisher exact test was used when it was appropriate for comparisons between categorical variables. The accuracy of clinical performance of p16^INK4a^/Ki-67 DS results for the diagnosis of CIN2+ was evaluated as sensitivity, specificity, and positive and negative predictive values, considering histomorphology as a gold standard.

## 3. Results

This study included 169 women from nine medical facilities, with a mean age of 37.4 years. Out of 169 women, who met the inclusion criteria, 7 had cytology smears unsatisfactory for evaluation. 162 cytology material was stained with p16^INK4a^/Ki-67 DS and with Pap stain in parallel. Out of all women, 29 histopathology results were available. Out of 162 immunocytochemistry cases, 16 (9.8%) were p16^INK4a^/Ki-67 DS positive.

In our study, 80.9% of all women had different types of cytologic abnormality (SIL and ASC) based on the Pap test result and categorization, or results were distributed as follows: NILM 31 (19.1%); ASC-US 27 (16.7%); ASC-H 5 (3.1%); LSIL 93 (57.4%); and HSIL 6 (3.7%). Out of 131 (80.9%) abnormal Pap test results, TBS categories were identified as follows: ASC-US 27 (20.6%); ASC-H 5 (3.8%); LSIL 93 (70.9%); and HSIL 6 (4.6%) (Table 1). Out of all abnormal Pap test results, the positive rate of p16^INK4a^/Ki-67 DS was 12.2% (Table 2).

Out of 29 histopathology results, 11 were CIN2+ out of which 2 case were equivocal for CIN2/CIN3, and 2 were CIN2 and 7 were CIN3; 5 results were CIN1, out of which 1 result was equivocal for CIN1/metaplasia; 5 results were chronic lymphocytic cervicitis; and 7 results were with normal histomorphology (Tables 3 and 4).

Out of 29 tissue paraffin block, 3 cases with the equivocal histopathological diagnosis was used for p16 IHC staining.

Of the 16 women, who had a positive p16^INK4a^/Ki-67 DS cervical cytology, 11 of them had histologically CIN 2+ results, and 1 woman had a histologically CIN1 result. Positive p16^INK4a^/Ki-67 DS result of cervical smear was not detected in any other category of histomorphology results (Table 4). The consensus among pathologists for p16^INK4a^/Ki-67 DS results was 100%.

The statistical analysis revealed that positive p16^INK4a^/Ki-67 DS, irrespective of the morphology of stained cells, of the cervical smear, and CIN2+ histology results of cervical biopsy have statistically significant dependence (*p* = 2.5 × 10^−6^ or *p* < <0.01). The p16^INK4a^/Ki-67 DS of the cervical smear and H&E histopathology cross-tabulation and results of statistical analysis are given in Tables 5 and 6.

Sensitivity, specificity, positive predictive value (PPV), and negative predictive value (NPV) of positive p16^INK4a^/Ki-67 DS, irrespective of the morphology of stained cells, to detect histologic CIN2+ lesion, and considering histopathology as the gold standard were 100%, 89%, 85%, and 100% (*p* = 2.5 × 10^−6^ < <0.01; Table 7). Interpretative variability was not observed between cytotechnologists and pathologists in the assessment of p16^INK4a^/Ki-67 dual biomarker expression.

In our study, the analysis of Pap test and histopathology results revealed a sensitivity, specificity, PPV, and NPV of 9%, 100%, 85%, and 64%, respectively (*p* = 0.6 > 0.05) for Pap test in detecting CIN2+ (Table 7). The sensitivity and specificity of the Pap test in the LSIL group to detect CIN1 was 18% and 61%, respectively.

Based on our data, no statistically significant dependence was found between p16^INK4a^/Ki-67 DS results and Pap test results. We observed different immunostaining results within the same category of Pap test results, as shown in Table 3.

The most frequent and rarest histopathological results were CIN2+ and chronic lymphocytic cervicitis, respectively (Table 1). The most frequent and rarest Pap cytological results were LSIL and HSIL, respectively (Table 1). Positive rate p16^INK4a^/Ki-67 DS among all histopathology results given in Table 4.

For one case with equivocal CIN1/metaplasia, there was no p16 IHC staining observed. However, for two cases with equivocal CIN2/CIN3, there were continuous staining of the entire epithelial thickness. Therefore, the first equivocal case was assessed as non SIL, whereas the second and third equivocal cases were assessed as CIN3 HSIL, following the criteria of LAST project. The dual p16^INK4a^/Ki67 immunocytostaining was negative for the case with negative p16 IHC, which had LSIL as the Pap test result. On the other hand, the dual p16^INK4a^/Ki67 immunocytostaining was positive for both cases with positive p16 IHC. Although we were unable to statistically correlate p16 IHC, H&E histopathology, and p16^INK4a^/Ki-67 DS in our study, we did observe the immunostaining results for three equivocal cases with corresponding H&E histology, p16 IHC, and p16^INK4a^/Ki-67 DS (Table 8). It is worth noting that there was 100% consensus among pathologists regarding the p16 IHC staining for all equivocal H&E histology cases.

The Pap cytology results of 10 women with DS-negative cervical cytology, who had a mean age of 41.5 years at enrollment were assessed after a median follow-up time of 6.5 years (ranging from 3 to 10 years). The results are provided in Table 9.

## 4. Discussion

Our study reveals the first results of p16^INK4a^/Ki-67 DS of cervical smear in Georgia.

Due to the abundance of abnormal Pap test results in cytological screening, we were interested in the expression peculiarities of the of p16^INK4a^ and K67 biomarkers in cervical smears. This research allowed us to study the features of the of p16^INK4a^/Ki-67 dual biomarker expression in the cervical cytological screening material and compare the accuracy of the immunocytochemical methodology and the Pap test in detecting high-grade dysplasia of the cervix.

Our study revealed a statistically significant correlation between p16^INK4a^/Ki-67 DS in cervical cytology and histopathology results of high-grade dysplasia of the uterine cervix (*p* = 2.5 × 10^−6^ or *p* < <0.01); In the study sensitivity of p16^INK4a^/Ki-67 DS cytology, regardless of the morphology of stained cells, in detecting histologic CIN2+ (CIN2 and CIN3) was 100% (Table 10), which is comparable with several other studies [31, 32, 34]. Gajsek et al. reported a sensitivity of 88.1% for p16^INK4a^/Ki-67 DS in detecting CIN2+ [30]. Ikenberg et al. reported an 86.7% sensitivity for p16^INK4a^/Ki-67 dual-stained cytology in detecting CIN2+ [31]. Luttmer et al. reported that the sensitivity of p16^INK4a^/Ki-67 dual-stained cytology for ≥CIN3 (93.8%) did neither differ significantly from Pap cytology (87.7%; ratio 1.07 and 95% confidence interval (CI): 0.97–1.18) nor from Pap cytology combined with HPV16/18 genotyping (95.1%; ratio 0.99 and 95% CI: 0.91–1.07) [32].

Our study revealed a sensitivity of 100% and specificity of 91% for detecting CIN2+ in LSIL cytology group, whereas the sensitivity and specificity in ASC (ASC-US and ASC-H) cytology group were 100 and 86%, respectively. Schmidt et al. reported sensitivity rates of 92.2% (ASC-US) and 94.2% (LSIL) for p16^INK4a^/Ki-67 dual-stained cytology in biopsy-confirmed CIN2+, with specificity rates of 80.6% (ASC-US) and 68.0% (LSIL) [33].

In our study, specificity, PPV, NPV, and accuracy of p16^INK4a^/Ki-67 DS in detecting histologic CIN2+ (CIN2 and CIN3) were 89%, 85%, 100%, and 93%, respectively (Table 10). Gajsek et al. reported a specificity of 65.2% for p16^INK4a^/Ki-67 DS in detecting CIN2+, with a PPV of 44.6% and an NPV of 94.5% [30]. Luttmer et al. reported that the specificity of p16^INK4a^/Ki-67 dual-stained cytology for ≥CIN3 (51.2%) was significantly higher than that of Pap cytology (44.9%; ratio 1.14 and 95% CI: 1.01–1.29) and Pap cytology combined with HPV16/18 genotyping (25.8%; ratio 1.99 and 95% CI: 1.68–2.35) [32].

According to our study, the prevalence of cytologic abnormalities (SIL and ASC) based on Pap test results is 80.9%. The prevalence of LSIL, ASC-US, ASC-H, and HSIL in our study was 57.4%, 16.7%, 3.1%, and 3.7%, respectively. The distribution of categories in Pap test results varies among different studies: for ASC-US it varies between 4.3% and 65%; for ASC-H, it ranges from 2% to 20.9%; for LSIL, it ranges from 2% to 27%; and for HSIL, it ranges from 0.5% to 15.6% [34–38]. Arslan et al. reported in their study that the prevalence of abnormal Pap cytology was 4.7%. Among these cases, the frequency of initial abnormal cytology was 65%, 27%, 3.4%, 2.4%, 1.9%, and 0.3% for ASC-US, LSIL, ASC-H, HSIL, typical AGC, and invasive cancer, respectively [38].

In our study, sensitivity, specificity, PPV, NPV and accuracy of conventional Pap test to detect CIN2+ lesions were 9%, 100%, 85%, 64% and 66%, respectively (Table 7). These data vary among studies (Table 10) [31, 34–38]. Nkwabong et al. reported in their study that the sensitivity, specificity, PPV, and NPV of Pap smear were 55.5%, 75%, 88.2%, and 33.3%, respectively [7].

In our study, out of 93 LSIL cytology cases 11 cases were DS-positive, and out of 32 ASC cases, 3 cases were DS-positive. Twenty-seven women out of 162 chose to undergo immunocytochemistry co-testing due to dissatisfaction with the result of the previous Pap test. The interval for re-testing in these cases was 1–2 months. Among these 27 women, re-testing of 18 women revealed different Pap test results compared with the previous ones. Our study showed a high consensus on immunostaining results among pathologists.

The limitations of our study were the sample size and possible selection bias. The study group consisted of participants in opportunistic cervical cancer screening who had a history of abnormal Pap test results. DS immunocytochemical testing of cervical material was conducted in three laboratories between March 2011 and December 2013 in Georgia. The following factors might have affected the sample size and selection bias: (1) immunocytochemical examination was offered to all women mentioned above. However, not everyone could undergo nonbudgetary DS examination despite having repeated abnormal cytology results or having nonidentical cytology results from several medical centers. Likely, not cost effectiveness seems appear one of the major reasons of fragmented service for medical centers as well, as only 169 immunocytochemistry tests were conducted over a 2.5-year period. Our study includes the results of all the mentioned cases (seven was unsatisfactory). (2) The cervical biopsy was performed based on the gynecologist's recommendation. Not all woman had the offer for biopsy according to the cervical cancer screening protocol, and not all women recommended for biopsy underwent the procedure. In some cases, cryodestruction of cervical lesions took place without collecting histological material. Therefore, a biopsy was performed for 29 women, from which 29 H&E histopathology and 3 p16 IHC tests were conducted. Unfortunately, after the mentioned period, we did not have the opportunity to collect additional materials in Georgia. Despite these limitations, we decided to publish the analysis based on the materials we have collected.

The strength of the study is that the collection of material and examination were conducted simultaneously with pathomorphological and immunostaining methodologies. Although we had a limited amount of study material, all women with H&E stained histopathology CIN2+ results and all women with p16 IHC positive results had p16^INK4a^/Ki-67 positive cytology. The results of further randomized trials are important.

In our study, positive DS cytology results were found in two CIN1 histopathology cases out of five CIN1 cases. Since our research includes the detection of p16^INK4a^/Ki-67 DS cells regardless of their morphology in cytological screening, it is interesting for us to study cytologic morphological features of DS cells in low-grade and high-grade CIN.

Unfortunately, we lacked the opportunity to analyze the results of a follow-up examination with p16^INK4a^/Ki-67 DS, especially in woman with positive DS results. However, we were able to analyze follow-up testing based on Pap test results of 10 women from our research group (mean age at initial screening was 41.5 years), who had DS-negative results during the primary testing. Pap cytology results were negative for intraepithelial lesion or malignancy (NILM) in all of them after a 3–10 years interval (median follow-up time of 6.5 years). The initial Pap cytological results in eight cases were LSIL, and two cases were NILM. Although we do not have statistically significant results of the follow-up examination, the results could be promising in terms of reflecting on the screening interval for women with normal cervical cytology. The results of further randomized trials are important. Clarke et al. reported that women with DS-negative findings had significantly lower 5-year risks of ≥CIN2 development compared with women with normal cytology (8.5%; 95% CI, 6.5%–11.1% vs. 12.3%; 95% CI, 9.8–15.4%; *p* = .04). In DS-negative women, the risks of both ≥CIN2 and ≥CIN3 remained below the colposcopy referral threshold for all 5 years, crossing the 1-year return threshold at 3 years [39].

Based on our results and also on results of existing studies, we can conclude that p16^INK4a^/Ki-67 DS cytology is of great significance in screening of cervical cancer and triaging of precancerous lesions.

Integration of biomarkers in cytology may be key for pathologists to solve the problems related to the interpretation difficulties associated with overlapping neoplastic and non-neoplastic squamous changes in cytology. DS cytology might be also significant tool to minimize the psychological and economic burden of the cervical screening. Despite of the high cost of single testing, p16^INK4a^/Ki-67 dual immunocytochemistry could be cost-effective in long term perspectives, as it has been shown to be helpful in triaging of ASC/LSIL categories and reducing the health and financial problems caused by underling invasive cancer, as well as by unnecessary invasive tests. In low-income countries, where screening is opportunistic, and there is low capacity of cytology referral centers, p16^INK4a^/Ki-67 DS can be considered as a tool for quality control of cytology screening.

## Figures and Tables

**Table 1:** Frequency of p16^INK4a^/Ki-67 DS, pap test, and histopathologic results.

Conventional pap cytology	NILM	ASC-US	ASC-H	LSIL	HSIL	Total
Conventional pap cytology	31 (NILM)	27 (ASC-US)	5 (ASC-H)	93 (LSIL)	6 (HSIL)	162
p16^INK4a^/Ki-67 DS	0	1	2	11	2	16
Histomorphology total						29
CIN1			3	3		6
CIN1/metaplasia equivocal				1		1
CIN2				2		2
CIN3		1		5	1	7
CIN2/CIN3 equivocal				2		2
Chronic lymphocytic cervicitis				5		5
Normal histology		4		3		7

**Table 2:** Distribution of conventional Pap test and p16^INK4a^/Ki-67 DS results.

Pap test p16^INK4a^/Ki-67 DS	NILM (31)	ASC-US (27)	ASC-H (5)	LSIL (93)	HSIL (6)	Total (162)
p16^INK4a^/Ki-67 DS	0	1	2	11	2	16
p16^INK4a^ staining	4	3	0	6	2	15
Ki-67 staining	3	0	0	10	0	13
p16^INK4a^ and Ki-67 staining independently	7	13	1	23	1	45
No staining at all	17	10	2	43	1	73

**Table 3:** Distribution of Pap test and H&E histology results.

	NILM	ASC-US	ASC-H	LSIL	HSIL	Total
CIN2+ (11 case)	0	1		9	1	11 (37.9%)
CIN1 (5 case)	0		3	2		5 (17.2%)
CIN1/metaplasia (1 case)	0			1		1 (3.6%)
Chronic lymphocytic cervicitis (5 case)	0			5		5 (17.2%)
Normal histology (7 case)	0	4		3		7 (24.1%)
Total (29 case)	0	5 (17.2%)	3 (10%)	20 (69%)	1 (3.4%)	29

**Table 4:** p16^INK4a^/Ki-67 DS and H&E histopathological results.

H&E histology	Positive p16^INK4a^/Ki-67 DS
CIN2+ (11)	11 (100%)
CIN1 (5)	2 (40%)
CIN1/metaplasia (1)	0
Chronic lymphocytic cervicitis (5)	0
Normal (7)	0
Total (29)	13

**Table 5:** p16^inka4a^/Ki67 DS H&E cervical histopathology cross tabulation.

			H&E stained cervical histopathology		Total
			No CIN2+	CIN2+ (CIN2 and CIN3)	
p16inka4a/Ki67DS	No	Count	16	0	16
		% within p16inka4a/Ki67DS group	100.0%	0.0%	100.0%
		% within H&E stained cervical histopathology group	88.9%	0.0%	55.2%
	Yes	Count	2	11	13
		% within p16inka4a/Ki67DS group	15.4%	84.6%	100.0%
		% within H&E stained cervical histopathology group	11.1%	100.0%	44.8%
Total		Count	18	11	29
		% within p16inka4a/Ki67 DS group	62.1%	37.9%	100.0%
		% within H&E stained cervical histopathology group	100.0%	100.0%	100.0%

**Table 6:** Results of the statistical analysis.

	Value	df	Asymp. Sig. (two-sided)	Exact sig. (two-sided)	Exact sig. (one-sided)
Pearson chi-square	21.812^a^	1	3.00718842851831 × 10^−6^		
Continuity correction^b^	18.366	1	0.0000182282005969676		
Likelihood ratio	27.334	1	1.71203259956547 × 10^−7^		
Fisher's exact test				2.25451184182345 × 10^−6^	2.25451184182345 × 10^−6^
Linear-by-linear association	21.060	1	4.45163412822054 × 10^−6^		
*N* of valid cases	29				

^a^One cell (25.0%) has expected count less than 5. The minimum expected count is 4.93.

^b^Computed only for a 2 × 2 table.

**Table 7:** Sensitivity, specificity, PPV, NPV, and accuracy of p16^INK4a^/Ki-67 DS and Pap test.

Groups Women aged 21–65 years (cytology, *n* = 162; histopathology, *n* = 29).	Sensitivity (%) (95% CI)	Specificity (%) (95% CI)	PPV (%)	NPV (%)	Accuracy (%)
p16^INK4a^/Ki-67 DS	100	89	85	100	93
Pap cytology	9	100	85	64	66

**Table 8:** Distribution of H&E histopathology and immunostaining results.

Equivocal H&E histopathology cases	Dual p16^INK4a^/Ki-67 immunocytochemistry positive	p16^INK4a^ IHC positive	p16^INK4a^ IHC negative
CIN1/metaplasia; 1 case	0	0	1
CIN2/ CIN 3; 2 cases	2	2	0

**Table 9:** Median 6.5 years follow-up cytology results in DS negative women.

Time of initial screening	Age at initial screening (years)	Results of p16^inka^4/Ki67 DS during initial screening	Results of pap test during initial screening	Time of follow-up screening	Results of follow-up Pap test screening
2011	30	Negative	LSIL	2014	NILM
2011	34	Negative	LSIL	2015	NILM
2012	37	Negative	NILM∗	2016	NILM
2011	39	Negative	NILM	2017	NILM
2011	39	Negative	LSIL	2018	NILM
2012	40	Negative	LSIL	2018	NILM
2011	41	Negative	LSIL	2019	NILM
2012	42	Negative	LSIL	2020	NILM
2011	50	Negative	LSIL	2021	NILM
2011	63	Negative	LSIL	2021	NILM

NILM∗, negative for intraepithelial lesion or malignancy.

**Table 10:** Sensitivity, specificity, PPV, and NPV of p16^INK4a^/Ki-67 DS for detection of CIN2+ in various study.

Study	Test on cervical smear for detection CIN2+ lesions	Sensitivity (%) (95% CI)	Specificity (%) (95% CI)	PPV (%)	NPV (%)
Gajsek et al. [30]	p16^INK4a^/Ki-67 DS	88.1	65.2	44.6	94.5
Ikenberg et al. [31]		86.7	95.2	15.6	99.9
Luttmer et al. [32]		93.8	51.2	29.9%	2.6
Nkwabong et al. [38]	Pap test	55.5	75	88.2	33.3
Ikenberg et al. [31]		68.5	95.4	13.3	99.7

## Data Availability

The authors confirm that all data underling the results are available as part of the article and no additional source of data are required.
